# Genomic characterization of plant cell wall degrading enzymes and *in silico* analysis of xylanses and polygalacturonases of *Fusarium virguliforme*

**DOI:** 10.1186/s12866-016-0761-0

**Published:** 2016-07-12

**Authors:** Hao-Xun Chang, Craig R. Yendrek, Gustavo Caetano-Anolles, Glen L. Hartman

**Affiliations:** Department of Crop Sciences, University of Illinois, Urbana, IL 61801 USA; Institute for Genomic Biology, Urbana, IL 61801 USA; USDA–Agricultural Research Services, Urbana, IL 61801 USA; National Soybean Research Center, University of Illinois, 1101 W. Peabody Dr., Urbana, IL 61801 USA

**Keywords:** Soybean, Sudden death syndrome (SDS), *Fusarium virguliforme*, Plant cell wall degrading enzymes (PCWDEs), Transgenic soybeans, Xylanases, Polygalacturonase, PCWDE inhibitor proteins (PIPs)

## Abstract

**Background:**

Plant cell wall degrading enzymes (PCWDEs) are a subset of carbohydrate-active enzymes (CAZy) produced by plant pathogens to degrade plant cell walls. To counteract PCWDEs, plants release PCWDEs inhibitor proteins (PIPs) to reduce their impact. Several transgenic plants expressing exogenous PIPs that interact with fungal glycoside hydrolase (GH)11-type xylanases or GH28-type polygalacturonase (PG) have been shown to enhance disease resistance. However, many plant pathogenic *Fusarium* species were reported to escape PIPs inhibition. *Fusarium virguliforme* is a soilborne pathogen that causes soybean sudden death syndrome (SDS). Although the genome of *F. virguliforme* was sequenced, there were limited studies focused on the PCWDEs of *F. virguliforme*. Our goal was to understand the genomic CAZy structure of *F. viguliforme*, and determine if exogenous PIPs could be theoretically used in soybean to enhance resistance against *F. virguliforme.*

**Results:**

*F. virguliforme* produces diverse CAZy to degrade cellulose and pectin, similar to other necrotorphic and hemibiotrophic plant pathogenic fungi. However, some common CAZy of plant pathogenic fungi that catalyze hemicellulose, such as GH29, GH30, GH44, GH54, GH62, and GH67, were deficient in *F. virguliforme*. While the absence of these CAZy families might be complemented by other hemicellulases, *F. virguliforme* contained unique families including GH131, polysaccharide lyase (PL) 9, PL20, and PL22 that were not reported in other plant pathogenic fungi or oomycetes. Sequence analysis revealed two GH11 xylanases of *F. virguliforme*, FvXyn11A and FvXyn11B, have conserved residues that allow xylanase inhibitor protein I (XIP-I) binding. Structural modeling suggested that FvXyn11A and FvXyn11B could be blocked by XIP-I that serves as good candidate for developing transgenic soybeans. In contrast, one GH28 PG, FvPG2, contains an amino acid substitution that is potentially incompatible with the bean polygalacturonase-inhibitor protein II (PvPGIP2).

**Conclusions:**

Identification and annotation of CAZy provided advanced understanding of genomic composition of PCWDEs in *F. virguliforme*. Sequence and structural analyses of FvXyn11A and FvXyn11B suggested both xylanases were conserved in residues that allow XIP-I inhibition, and expression of both xylanases were detected during soybean roots infection. We postulate that a transgenic soybean expressing wheat XIP-I may be useful for developing root rot resistance to *F. virguliforme*.

**Electronic supplementary material:**

The online version of this article (doi:10.1186/s12866-016-0761-0) contains supplementary material, which is available to authorized users.

## Background

Inactivation of pathogen plant cell wall degrading enzymes (PCWDEs) is one of the strategies that plants employ to prevent infection. Several plant-derived extracellular PCWDEs inhibitor proteins (PIPs) were reported to not only reduce PCWDEs activities but also trigger defense response upon recognition of PCWDEs [1–3]. The importance of PIPs in plant defense has been demonstrated in transgenic plants expressing exogenous PIPs that show enhanced biotic resistance. For example, wheat xylanase inhibitors, such as xylanase inhibitor protein I (XIP-I) and *Triticum aestivum* xylanase inhibitor III (TAXI-III), have been shown to inhibit fungal GH11 xylanases [3–5]. Similarly, transgenic wheat with TAXI-III increased resistance to necrosis and head blight caused by *Fusarium graminearum* [6–8]. Another well-studied example is the polygalacturonase (PG) inhibitor proteins (PGIP), a leucine-rich repeat protein of plants that interact with fungal GH28 PG [2, 9]. Several transgenic plants expressing exogenous PGIPs have been shown to increase resistance against a broad spectrum of pathogens [10–16]. The mechanism of enhanced resistance in PGIP-transgenic plants has been recently demonstrated. In vivo expression of chimeric PGIP-PG in Arabidopsis showed that PGIP-PG interaction induced the production of oligogalacturonides, which serves as a damage-associated molecular mechanism to stimulate resistance [17].

Soybean sudden death syndrome (SDS), which is caused by a soilborne fungus *F. virguliforme*, is responsible for annual losses around US$190 million [18]. Breeding for SDS resistance is difficult because the interaction between *F. virguliforme* and soybean is quantitative [19]. Instead, it has been suggested that transgenic approaches may be suitable to manage SDS, and transgenic soybeans expressing exogenous toxin-specific antibody has been shown to reduce SDS foliar symptoms [18, 20]. However, symptoms caused by *F. virguliforme* include not only foliar symptoms but also root rot and vascular discoloration [18]. Soybeans that exhibit partial root resistance have been shown to have up-regulated genes involved in plant cell wall enhancement upon root infection by *F. virguliforme* [21]. Differences in root susceptibility of soybean genotypes also showed different expression patterns of genes involved in plant cell wall synthesis [22]. These studies indicated that plant cell wall modification maybe involved in resistance against *F. virguliforme*, which highlights the possibility of using transgenic soybeans that express exogenous PIPs to prevent and/or slow fungal colonization of soybean roots. Therefore, an *in silico* study would be useful before embarking in a time-consuming transgenic project, as it would be important to know if *F. virguliforme* secrets compatible PCWDEs to the transgenic exogenous PIPs during infection.

Although the genome of *F. virguliforme* has been published [23], genomic structure of PCWDEs remains uncharacterized. In this study, we annotated PCWDEs in the *F. virguliforme* genome, and further focused on the orthologous GH11 xylanases and GH28 PGs of *F. virguliforme*. The goal was to understand the genomic PCWDEs structure of *F. virguliforme* and to evaluate if orthologous GH11 xylanases and GH28 PGs of *F. virguliforme* have potential to serve as targets for exogenous PIPs produced by transgenic soybeans.

## Results and discussion

### Identification of carbohydrate-active enzymes (CAZy) in the genome of *F. virguliforme*

CAZy are proteins with polysaccharide-degrading enzymatic activities on polysaccharides [24, 25]. We identified 629 putative genes that encode CAZy in the genome of *F. virguliforme* (Additional file 1: Table S1). Of the six CAZy classes, carbohydrate esterases (CE), glycoside hydrolases (GH), and polysaccharide lyases (PL) are PCWDEs. There were 66, 292, and 28 genes belonging to the CE, GH, and PL classes, respectively (Table 1). Three other classes with indirect roles on degrading carbohydrates are auxiliary activity (AA), carbohydrate-binding module (CBM), and glycosyl-transferase (GT). There were with 96, 31, and 116 genes identified in the AA, CBM, and GT classes, respectively (Table 2).

**Table 1:** Plant cell wall degrading enzymes (CE, GH and PL classes) of *Fusarium virguliforme*

CAZy family	Substrate	Annotation	EC number	Copy number
CE1	Hemicellulose (xylan)	Acetyl xylan esterase	3.1.1.72	34
		Feruloyl esterase	3.1.1.73	
CE2	Hemicellulose (xylan)	Acetyl xylan esterase	3.1.1.72	1
CE3	Hemicellulose (xylan)	Acetyl xylan esterase	3.1.1.72	5
CE4	Hemicellulose (xylan)	Acetyl xylan esterase	3.1.1.72	7
CE5	Hemicellulose (xylan)	Acetyl xylan esterase	3.1.1.72	7
	Cutin	Cutinase	3.1.1.74	
CE8	Pectin (homogalacturonan)	Pectin methylesterase	3.1.1.11	2
CE9	Polysaccharides	N-acetylglucosamine 6-phosphate	3.5.1.25	1
		Deacetylase	3.5.1.80	
CE12	Hemicellulose	Acetyl pectin esterase	3.1.1.72	3
	Pectin (homogalacturonan, rhamnogalacturonan I)	Pectin acetylesterase	3.1.1.-	
CE14	Polysaccharides	N-acetylglucosaminylphosphatidy-linositol deacetylase	3.5.1.89	1
CE16	Polysaccharides	Acetylesterase	3.1.1.6	5
GH1	Cellulose	β-glucosidase	3.2.1.21	5
	Hemicellulose (xylan, xyloglucan)	β-xylosidase	3.2.1.37	
	Pectin (rhamnogalacturonan I)	β-galactosidase	3.2.1.23	
GH2	Hemicellulose (xylan, xyloglucan, galactomannan)	β-mannosidase	3.2.1.25	8
	Pectin (rhamnogalacturonan I)	β-glucuronidase	3.2.1.31	
GH3	Cellulose	β-glucosidase	3.2.1.21	22
	Hemicellulose	β-xylosidase	3.2.1.37	
	(xylan, xyloglucan)		3.2.1.74	
	Pectin	exo-β-1,4-glucanase		
GH5	Cellulose	endo-β-1,4-glucanase	3.2.1.4	15
	Hemicellulose (galactomannan)	endo-β-1,4-xylanase	3.2.1.8	
	Pectin (rhamnogalacturonan I)	exo-β-1,4-glucanase	3.2.1.74	
GH6	Cellulose	endo-β-1,4-glucanase	3.2.1.4	1
		cellobiohydrolase	3.2.1.91	
GH7	Cellulose	endo-β-1,4-glucanase	3.2.1.4	3
		Cellobiohydrolase	3.2.1.176	
GH10	Hemicellulose (xylan)	endo-β-1,4-xylanase	3.2.1.8	3
GH11	Hemicellulose (xylan)	endo-β-1,4-xylanase	3.2.1.8	3
GH12	Cellulose	endo-β-1,4-glucanase	3.2.1.4	6
	Hemicellulose (xyloglucan)	Xyloglucanase	3.2.1.151	
GH13	Polysaccharides	α-amylase	3.2.1.1	7
GH15	Polysaccharides	Glucoamylase	3.2.1.3	3
GH16	Hemicellulose	Xyloglucanase	3.2.1.151	19
GH17	Polysaccharides	endo-1,3-β-glucosidase	3.2.1.39	5
GH18	Polysaccharides	Chitinase	3.2.1.14	22
		endo-β-N-acetylglucosaminidase	3.2.1.96	
GH20	Polysaccharides	β-hexosaminidase	3.2.1.52	1
GH23	Polysaccharides	Chitinase	3.2.1.14	2
		Lysozyme type G	3.2.1.17	
GH24	Polysaccharides	Lysozyme	3.2.1.17	2
GH27	Hemicellulose (xylan, xyloglucan, galactomannan)	α-galactosidase	3.2.1.22	1
		α-N-acetylgalactosaminidase	3.2.1.49	
GH28	Pectin (homogalacturonan, rhamnogalacturonan I)	Polygalacturonase	3.2.1.15	8
GH31	Hemicellulose (xyloglucan)	α-xylosidase	3.2.1.177	9
GH32	Sucrose	Invertase	3.2.1.26	4
GH33	Oligosaccharides	exo-α-sialidase	3.2.1.18	1
GH35	Hemicellulose (xylan, xyloglucan, galactomannan)	β-galactosidase	3.2.1.23	4
	Pectin (rhamnogalacturonan I)	exo-β-1,4-galactanase	3.2.1.-	
GH36	Hemicellulose (xylan, xyloglucan, galactomannan)	α-galactosidase	3.2.1.22	2
		α-N-acetylgalactosaminidase	3.2.1.49	
GH37	Trehalose	α,α-trehalase	3.2.1.28	2
GH38	Oligosaccharides	α-mannosidase	3.2.1.24	1
GH43	Hemicellulose (xylan)	β-xylosidase	3.2.1.37	26
	Pectin (rhamnogalacturonan I)	α-L-arabinofuranosidase	3.2.1.55	
GH45	Cellulose	endo-β-1,4-glucanase	3.2.1.4	2
GH47	Oligosaccharides	α-mannosidase	3.2.1.113	10
GH51	Cellulose	endo-β-1,4-glucanase	3.2.1.4	2
	Hemicellulose (xylan,xyloglucan)	β-xylosidase	3.2.1.37	
GH53	Pectin (rhamnogalacturonan I)	endo-β-1,4-galactanase	3.2.1.89	1
GH55	Polysaccharides	endo-1,3-β-glucosidase	3.2.1.39	6
GH63	Oligosaccharides	α-glucosidase	3.2.1.106	1
GH64	Polysaccharides	endo-1,3-β-glucosidase	3.2.1.39	2
GH71	Polysaccharides	α-1,3-glucanase	3.2.1.59	3
GH72	Polysaccharides	β-1,3-glucanosyltransglycosylase	2.4.1.-	3
GH74	Cellulose	endo-β-1,4-glucanase	3.2.1.4	2
	Hemicellulose (xyloglucan)	Xyloglucanase	3.2.1.151	
GH75	Polysaccharides	Chitosanase	3.2.1.132	2
GH76	Oligosaccharides	α-1,6-mannanase	3.2.1.101	8
GH78	Pectin	α-L-rhamnosidase	3.2.1.40	6
GH79	Pectin (rhamnogalacturonan I)	β-glucuronidase	3.2.1.31	1
GH81	Polysaccharides	endo-1,3-β-glucosidase	3.2.1.39	1
GH88	Polysaccharides	β-glucuronyl hydrolase	3.2.1.-	4
GH93	Pectin (rhamnogalacturonan I)	exo-α-L-1,5-arabinanase	3.2.1.-	3
GH95	Hemicellulose (xyloglucan)	α-1,2-L-fucosidase	3.2.1.63	2
GH99	Oligosaccharides	endo-α-1,2-mannosidase	3.2.1.130	1
GH105	Pectin	rhamnogalacturonyl hydrolase	3.2.1.172	4
GH109	Polysaccharides	α-N-acetylgalactosaminidase	3.2.1.49	26
GH114	Polysaccharides	endo-α-1,4-polygalactosaminidase	3.2.1.109	4
GH115	Hemicellulose (xylan)	Xylan α-1,2-glucuronidase	3.2.1.131	1
GH125	Oligosaccharides	exo-α-1,6-mannosidase	3.2.1.-	3
GH127	Oligosaccharides	β-L-arabinofuranosidase	3.2.1.185	4
GH128	Polysaccharides	endo-1,3-β-glucosidase	3.2.1.39	2
GH131	Cellulose	exo-β-1,3/1,4/1,6-glucanase	3.2.1.-	1
	Hemicellulose			
GH132	Polysaccharides	Activity on β-1,3glucan	–	2
PL1	Pectin (homogalacturonan)	Pectate lyase	4.2.2.2	11
PL3	Pectin	Pectate lyase	4.2.2.2	10
PL4	Pectin (rhamnogalacturonan I)	Rhamnogalacturonan lyase	4.2.2.-	4
PL9	Pectin	Pectate lyase	4.2.2.2	1
		Exopolygalacturonate lyase	4.2.2.9	
PL20	Pectin	endo-β-1,4-glucuronan lyase	4.2.2.14	1
PL22	Pectin	Oligogalacturonate lyase	4.2.2.6	1

**Table 2:** AA, CBM and GT classes of *Fusarium virguliforme*

CAZy family	Annotation	Copy number
AA1	Multicopper oxidases	4
AA2	Lignin peroxidase	4
AA3	glucose-methanol-choline (GMC) oxidoreductases	25
AA4	vanillyl-alcohol oxidase	5
AA5	radical-copper oxidases	2
AA6	1,4-benzoquinone reductases	2
AA7	Glucooligosaccharide oxidase	40
AA8	Iron reductase	2
AA9	copper-dependent lytic polysaccharide monooxygenases	12
CBM1	cellulose-binding	2
CBM4	cellulose-binding	1
CBM6	cellulose-binding	1
CBM13	cellulose-binding	2
CBM18	chitin-binding	2
CBM19	chitin-binding	2
CBM20	starch-binding	1
CBM21	starch-binding	2
CBM22	xylan-binding	4
CBM35	xylan-binding	1
CBM50	Peptidoglycan-binding (LysM domain)	5
CBM61	β-1,4-galactan-binding	4
CBM63	cellulose-binding	2
CBM67	L-rhamnose-binding	3
GT1	UDP-glucuronosyl-transferase	15
GT2	cellulose/chitin synthase	18
GT3	Glycogen synthase	1
GT4	Sucrose synthase	6
GT8	Lipopolysaccharide glucosyl-transferase	8
GT15	α-1,2-mannosyl-transferase	5
GT17	β-1,4-N-acetyl-glucosaminyl-transferase	1
GT20	α,α-trehalose-phosphate synthase	3
GT21	Ceramide β-glucosyl-transferase	3
GT22	Man6GlcNAc2-PP-Dol α-1,2-mannosyl-transferase	4
GT24	Glycoprotein α-glucosyl-transferase	1
GT26	β-N-acetyl-mannosaminuronyl-transferase	2
GT28	Digalactosyl-diacyl-glycerol- synthase	1
GT31	fucose-specific β-1,3-N-acetylglucosaminyl-transferase	2
GT32	α-1,6-mannosyl-transferase	7
GT33	chitobiosyl-diphosphodolichol β-mannosyl-transferase	1
GT34	α-1,2-galactosyl-transferase	3
GT35	Starch phosphorylase	1
GT39	Protein α-mannosylt-ransferase	3
GT48	1,3-β-glucan synthase	2
GT50	α-1,4-mannosyl-transferase	2
GT54	α-1,3-D-mannoside β-1,4-N-acetyl-glucosaminyl-transferase	1
GT57	α-1,3-glucosyl-transferase	2
GT58	Man5GlcNAc2-PP-Dol α-1,3-mannosyl-transferase	1
GT59	Glc2Man9GlcNAc2-PP-Dol α-1,2-glucosyl-transferase	1
GT62	α-1,2-mannosyl-transferase	3
GT64	Heparan α-N-acetyl-hexosaminyl-transferase	2
GT66	dolichyl-diphospho-oligosaccharide-protein glycotransferase	1
GT69	α-1,3-mannosyl-transferase	5
GT71	α-mannosyl-transferase	3
GT76	α-1,6-mannosyl-transferase	1
GT77	α-xylosyltransferase	1
GT90	glucuronoxylomannan/galactoxylomannan β-1,2-xylosyl-transferase	5

### Identification of putative cellulose-degrading enzymes in the genome of *F. virguliforme*

**Fig. 1:**
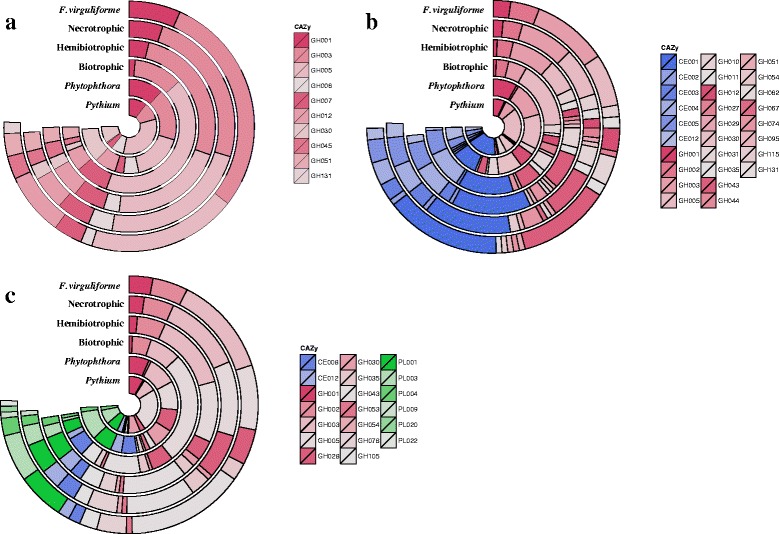
Comparison analysis for PCWDEs of *Fusarium virguliforme* that putatively target on different polysaccharides. Blue color indicates carbohydrate esterases (CE); red color indicates glycoside hydrolases (GH); and green color indicates polysaccharide lyases (PL). **a** CAZy with cellulase activity. GH1, GH3, and GH5 are universal PCWDEs that catalyze celluloses, hemicelluloses, and pectin. GH30 is common distributed in plant pathogenic fungi and abundant in oomycetes, but it was not found in the genome of *F. virguliforme*. Instead, GH131 was found only in the genome of *F. virguliforme*. **b** CAZy with hemicellulase activity. GH29, GH30, GH44, GH54, GH62, and GH67 are absent in the genome of *F. virguliforme*, but other functional redundant CAZy may complement the loss of these families. **c** CAZy with pectinase activity. *F. virguliforme* have most pectinases and unique PL9, PL20, and PL22 that only existed in *F. virguliforme* and close-related species *Nectaria haematococca*. In general, the genomic PCWDEs structure of *F. virguliforme* is similar to necrotrophic and hemibiotrophic pathogenic fungi

Cellulose is the most abundant component in plant cell walls, which results from the polymerization of glucose and the formation of a microfibril framework for other components to join [24, 26, 27]. Most cellulose-degrading enzymes are categorized within GH classes. GH1, GH3, and GH5 are prevalent PCWDEs that catalyze not only cellulose, but also hemicellulose and pectin (Table 1). Plant pathogenic oomycetes, and hemibiotrophic as well as necrotrophic fungi generally contain more GH1 degrading enzymes than biotrophic fungi. For example, the genome of *F. virguliforme* encodes five GH1 genes while most biotrophic fungi have none [28–30]. For enzymes in the GH3 family, *F. virguliforme*, hemibiotrophic and necrotrophic fungi, and *Phytophthora* species contain 8-38 genes compared to relatively fewer for biotrophic fungi and *Pythium* species (Fig. 1a). Endo- and exo-β-1,4-glucanases in the GH5 family are cellulose-degrading enzymes employed by both plant pathogenic fungi and oomycetes, and *F. virguliforme* has 15 GH family genes. In addition, *F. virguliforme* has one GH6 and three GH7 that not only have endo- and exo-β-1,4-glucanase but also cellobiohydrolase activity. GH12 encode cellulose/hemicellulose-degrading enzymes similar to GH3, which is common in *F. virguliforme*, plant pathogenic fungi and *Phytophthora* species but not in *Pythium* species. GH30 is dominant in oomycetes but not in plant pathogenic fungi, and none was found in *F. virguliforme* (Fig. 1a). On the other hand, GH45 and GH51 are fungi-specific degrading enzymes that have not been found in oomycetes [28, 30]. GH131 CAZy that encodes exo-β-1,3/1,6- and endo-1,4-glucanase was only found in *F. virguliforme*. In addition to GH families, some AA families, such as AA8 and AA9, have been reported to accelerate cellulose degradation. Instead of catalyzing carbohydrates, enzymes in the AA9 family (previously known as GH61) have copper-dependent lytic polysaccharide monooxygenase activity to assist degradation of lignocellulose [25, 31]. It has been suggested that plant pathogenic fungi have more AA9 genes than oomycetes [28–30], and 12 AA9 genes were found in *F. virguliforme* (Table 2).

### Identification of putative hemicellulose-degrading enzymes in the genome of *F. virguliforme*

Hemicellulose is composed of polymers such as xyloglucan, xylan and galactomannan, cross-links the cellulose microfibrils and provides strength to plant cell walls [24, 26, 27]. In addition to GH1, GH3, GH5, GH12, GH51, and GH131 that have both cellulose- and hemicellulose-degrading activities, GH2, GH10, and GH11 are important hemicellulose-degrading enzymes for plant pathogenic fungi including *F. virguliforme* (Table 1). However, these families are generally deficient in oomycetes, except GH10, which exists in *Phytophthora* species (Fig. 1b). GH29, GH30, GH44, GH54, GH62, and GH67 families are absent in the genome of *F. virguliforme*. A closely related species, *Nectria haematococca* (anamorph *Fusaium solani*), has no CAZy in the GH29 and GH30 either. Instead, *F. oxysporum* and *F. verticillioides* have at least two enzymes for each GH29 and GH30 [30]. Nevertheless, *F. virguliforme* contains two GH95 α-fucosidases that may have similar enzymatic activities to GH29 and GH30, which remove xyloses from xyloglucan [24]. *F. virguliforme* has no GH54 and GH62 that encode α-L-arabinofuranosidases, but *N. haematococca*, *F. oxysporum*, and *F. verticillioides* have at least one GH54 and one GH62 enzyme [30]. The function of GH54 and GH62 may be redundant to GH3, GH10, GH43, and GH51 [24], which could be found in the *F. virguliforme* genome (Table 2). Among these four families, GH43 is one of the largest CAZy that catalyzes both hemicellulose and pectin, and *F. virguliforme* has 26 genes. In addition, *F. virguliforme* has no GH44 or GH67 that are deficient in most plant pathogens. The loss of GH44 and GH67 may be complemented by GH74 and GH36, respectively, because both GH44 and GH 74 encode xyloglucanases while GH67 and GH36 both encode α-galactosidases (Table 2). Another group of CAZy active on hemicellulose is the CE class. CE1 is the most dominant hemicellulose-degrading family in plant pathogens, and in the case of *F. virguliforme*, 32 genes were found. CE families such as CE2, CE3, CE4, CE5, and CE12, were all identified in the genome of *F. virguliforme* as reported in other plant pathogens [28–30].

### Identification of putative pectin-degrading enzymes in the genome of *F. virguliforme*

Pectin, a polymer of mainly D-galacturonic acids, is the most divergent part of plant cell walls because of the different modifications on the side chains. Based on these modifications, pectin is categorized into subgroups like homogalacturonan and rhamnogalacturonan. Pectin forms a matrix between microfibrils to control the porosity and cohesion [24, 26, 27, 32, 33]. Besides the universal plant cell wall degrading families (GH1, GH3, and GH5) and the most well studied GH28 PGs, GH53 and GH78 are common in most hemibitrophic and necrotrophic fungi as well as *Phytophthora* species while GH105 is more abundant in plant pathogenic fungi than oomycetes (Fig. 1c). Except for the lack in GH30 and GH54 that have been discussed in the hemicellulose section, *F. virguliforme* has all the GH families that catalyze pectin. Some CAZy in the CE class, such as CE8 and CE12, allow degradation of pectin by removing methyl and acetyl groups from galacturonic acids, respectively. Both families are common in all plant pathogens including *F. virguliforme* but not *Pythium* species [29]. The PL class specializes in pectin degradation. PL1 and PL3 are the most dominant and common pectin lyases of plant pathogens. Similar to hemibitrophic and necrotrophic fungi and oomycetes, *F. virguliforme* has eleven PL1 and ten PL3 that are more abundant than bitrophic fungi [28–30]. In addition, PL4, PL9, PL20, and PL22 families were identified in the *F. virguliforme* genome (Fig. 1c). While PL4 is commonly distributed in plant pathogens, PL9, PL20, and PL22 were found only in *F. virguliforme* and *N. haematococca* [30].

### Evaluation of xylanases and PGs of *F. virguliforme* as PIPs targets

**Fig. 2:**
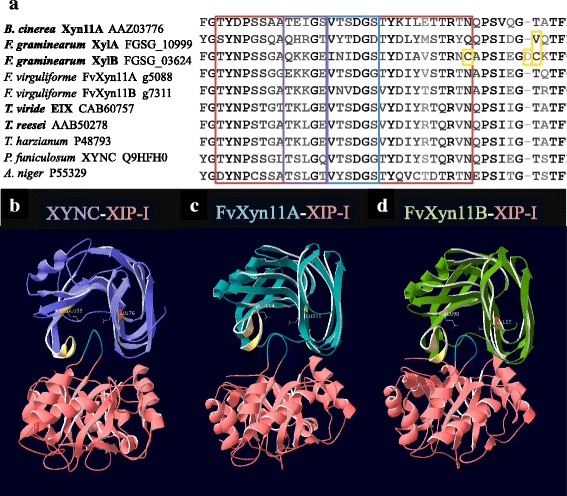
*In silico* analysis of GH11 xylanases of *Fusarium virguliforme*. **a** *F. graminearum* contains amino acid substitutions that allow GH11 xylanases to escape XIP-I inhibition, including a substitution of threonine (T) to valine (V) for XylA (yellow blocks); and substitutions of asparagine (N) to cysteine (C), an insert of aspartic acid (D), and a substitution of T to C for XylB (yellow blocks). However, FvXyn11A and FvXyn11B are conserved in this region. The red block circles a string of 30 amino acids reported to induce necrosis [63]. The purple block and blue block indicate previously reported conserved residues. The name of necrosis-inducing xylanases were bold [8, 63, 64]. **b** Salmon color represents XIP-I. Golden color represents conserved thumb region of each xylanase. Control model of XIP-I inhibits *Penicillium funiculosum* GH11 xylanase XYNC. XIP-I perfectly fills into the catalyzing groove between two essential catalyzing residues glutamic acid (E) at position 85 (E85) and E176 that mimics substrates of XYNC. **c** The interaction between FvXyn11A and XIP-I, where the corresponding residues E114 and E205 were shown. **d** The interaction between FvXyn11B and XIP-I, where the corresponding residues E98 and E189 were shown

GH11 xylanases and GH28 PGs have been successfully used as targets for transgenic plants expressing exogenous PIPs. However, GH11 xylanases and GH28 PGs of some *Fusarium* species can escape PIPs inhibition by amino acid substitution [34, 35]. Two GH11 xylanases, XylA (FGSG_10999) and XylB (FGSG_03624) of *F. graminearum*, have amino acid substitutions at the thumb region that allowed them to escape XIP-I binding (Fig. 2a) [4]. On the other hand, site-directed mutagenesis of lysine to glutamine of position 97 increased affinity of *F. verticillioides* PG to PvPGIP2 [35]; more importantly, a single substitution at the 261 position of *F. phyllophilum* PG (FpPG) from alanine to threonine significantly reduced FpPG affinity to PvPGIP2 [36]. Amino acid substitutions in these studies supported the variable response of PGIPs to PGs of different *Fusarium* species [36, 37].

**Fig. 3:**
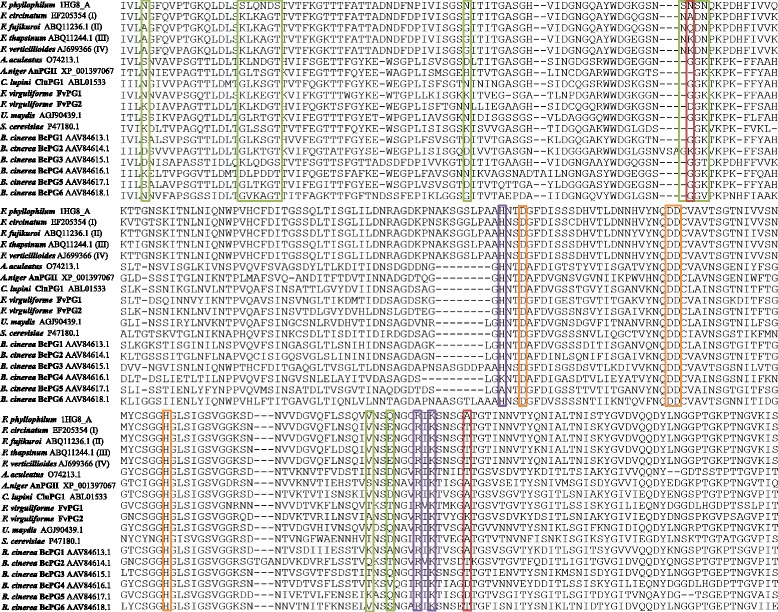
Sequence alignment of GH28 polygalacturonase of *Fusarium virguliforme* with other fungi. The green blocks circle polymorphic residues [37]; the purple blocks indicate essential residues for binding substrates [65]; the orange blocks circle indispensible residues for catalyzing substrates [66]; and the red blocks circle residues (position 97 in top panel and 261 in bottom panel) that were reported to affect PvPGIP2 inhibition [35, 36]

Two orthologous GH11 xylanases (FvXyn11A and FvXyn11B) and two orthologous PGs (FvPG1 and FvPG2) were identified for *F. virguliforme* (Table 3). Sequence analysis revealed that neither FvXyn11A nor FvXyn11B carry amino acid substitutions at the thumb region corresponding to XylA or XylB of *F. graminearum* (Fig. 2a). Protein-protein docking analysis was applied to further test the interaction between XIP-I and FvXyn11A as well as FvXyn11B. The results supported XIP-I forming inhibiting conformations with FvXyn11A and FvXyn11B in the same orientation to *Penicillium funiculosum* XYNC (Fig. 2b) [5]. In the case of FvPG1 and FvPG2, sequence alignment was uncertain at residue 97 because of the neighboring gaps (Fig. 3). However, because FvPG1 has an alanine at position 261 that is identical to *Colletotrichum lupin*e PG (CluPG1) and *Aspergillus niger* PG (AnPGII), we speculated that the affinity strength of FvPG1 to PvPGIP2 would be similar to CluPG1 and AnPG II [2, 36]. The replacement of the nonpolar alanine to the polar threonine dramatically reduces FpPG affinity to PvPGIP2 [36], so we speculated FvPG2 would be less inhibited by PvPGIP2 because the corresponding position of FvPG2 is a larger, positively charged lysine.

**Table 3:** Orthologous GH11 xylanases and GH28 polygalacturonases of *Fusarium virguliforme*

Gene Name^a^	Gene ID^a^	E value^a^	qRT-PCR Primer Sequence^a^	Amplicon^a^	Tm (°C)^b a^	AE^c a^	R^2a^
GH11 xylanase							
FvXyn11A	g5088	1.0 × 10^-77^	F- CTGTCATCACTACCCGAAGAC	104 bp	61.4	0.648	0.99
			R- CTGGGCTCGTTTGACTACAT		61.7		
FvXyn11B	g7311	6.0 × 10^-73^	F- TCAACGCCTGGAAGAATGTC	100 bp	62.2	0.702	1.00
			R- ACAGTCATGGTGGCAGAAC		61.9		
GH28 polygalacturonase							
FvPG1	g9942	5.0 × 10^-58^	F- AAACGGCGGCAAGAAGAA	91 bp	62.3	0.802	0.98
			R- GACGGGCGTGTTCTTGATATAG		62.3		
FvPG2	g13315	1.0 × 10^-68^	F- CCACTCTCTCAAGAACTCCAAC	110 bp	61.9	0.888	0.97
			R- CGAGATGAACATCGTAGACACC		61.9		
Reference gene							
FvEF1A	g4748	0.0	F- GGGTAAGGAGGAGAAGACTCA	98 bp	62.0	0.748	1.00
			R- CACCGCACTGGTAGATCAAG		62.0		

^a^E value for *F. virgulifrome* gene to query: *P.funiculosum* GH11 xylanase XYNC (Q9HFH0), *F. phyllophilum* FpPG (AAA74586.1), and *F. graminearum* EF1A (FGSG_08811.3) by BLASTN

^b^Tm of each primer was calculated by IDT Oligo Analyzer 3.1 with settings: 50 mM Na^+^, 3 mM Mg^2+^, 1 mM dNTP, and 200nM oligo

^c^Amplification efficiency

**Fig. 4:**
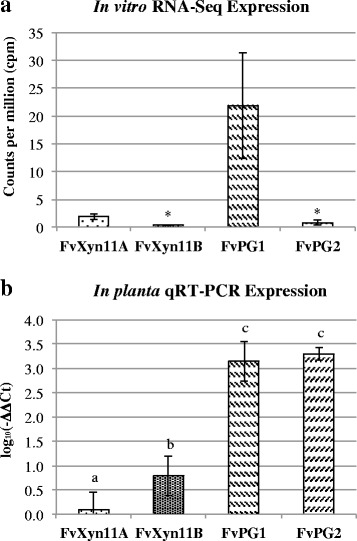
Expression comparison of FvXyn11B and FvPG2 in vitro and *in planta*. RNA of both conditions was extracted after 5 days post inoculation, from soybean dextrose broth and from soybean roots, respectively. **a** In vitro expression was indicated by counts per million (cpm) from a RNA-Seq data [38]. Asterisk indicated genes with raw counts below 1 cpm. **b** *In planta* expression indicated by log_10_(–ΔΔCt). Unlike in vitro condition, the expression of FvXyn11B increased and was significantly higher the FvXyn11A. The expression of FvPG2 was also increased to a level similar to FvPG1

FvXyn11A, FvXyn11B, FvPG1, and FvPG2 contained putative secretory peptides without trans-membrane domains. Moreover, their expressions were detectable during infection. Using an in vitro RNA-Seq dataset [38], we noticed FvXyn11B and FvPG2 were less active compared to FvXyn11A and FvPG1 in the in vitro condition (Fig. 4a). However, the expression of FvXyn11B and FvPG2 were significantly enhanced during root infection (Fig. 4b). It has been reported that PCWDEs of some *Fusarium* species displayed different expression patterns in different conditions. For example, two PGs of *F. oxysporum*, *pg1* and *pgx6*, expressed actively during root infection, and the double knockout mutants of *pg1* and *pgx6* compromised virulence [39]. In addition, differential expression of GH11 xylanases was also reported that XylB had higher expression than XylA at 5 day-post-inoculation [40].

## Conclusion

In this study, we advanced the understanding of CAZy and PCWDEs in the genome of *F. virguliforme* and *in silico* analysis supported the possibility of developing transgenic soybeans with exogenous PIPs to enhance SDS resistance. As a soybean pathogen, *F. virguliforme* may have undergone selection pressure to PGIPs produced by soybean. Our analysis revealed a putative PvPGIP2-escaping FvPG2 had higher expression during root infection than in the in vitro condition. This indicated that the use of transgenic PVPGIP2 might not be a preferable option. Instead, *F. virguliforme* should rarely encounter XIP-I because xylanase inhibitor proteins are more dominant in graminaceous plants such as wheat. Xylanases play important roles in fungal virulence. The endo-β-1,4-xylanase Xyn11A was shown to required for virulence in *Botrytis cinerea* [41] and xylanases knock-down mutants of *Magnaporthe oryzae* also caused less lesions compared to wild type *M. oryzae* [42]. Our results revealed FvXyn11A and FvXyn11B lack amino acid substitutions that would avoid XIP-I inhibition. Because XIP-I has been reported to inhibit both GH10 and GH11 xylanases [3–5], we consider XIP-I a better candidate since multiple targets of XIP-I may extend the persistence of the transgenic soybeans. In addition to inhibition of GH11 xylanase, XIP-I was reported to reduce cell death induced by necrosis-inducing xylanases, such as XylA and XylB of *F. graminearum* [8], and an orthologous XIP-I from coffee has been shown to inhibit the germination of soybean rust urediniospores [43]. Developing a transgenic soybean that expresses an exogenous XIP-I might not only reduce soybean rust infection but also enhance resistance against SDS.

## Methods

### Identification of CAZy in the *F. virguliforme* genome

The *Fusarium virguliforme* genome sequence (accession AEYB01000000) was downloaded from NCBI and is available at http://fvgbrowse.agron.iastate.edu [23]. Augustus was used to predict putative proteins in the genome and transcriptome with *F. graminearum* as a model organism using default parameters except for the minexonintronprob (=0.1) and minmeanexonintronprob (=0.4) [44]. CAZy domains were identified in genomes with dbCAN and a cutoff of E value of 10^-3^ [45]. When a gene contained a CBM with other CAZy classes, the gene was classified in the later classes. When redundancies were detected, classification was determined based on the lowest E value (Additional file 1: Table S1). Protein annotation was based on the CAZy database [25, 46]. The genomic CAZy structure of *F. virguliforme* was compared to other plant pathogenic fungi and oomycetes [28–30].

### *In silico* analyses of GH11 xylanases and GH28 PGs of *F. virguliforme*

Sequences of *P. funiculosum* GH11 xylanase XYNC [5] and *F. phyllophilum* GH28 FpPG [36] were used as queries to identify orthologous genes in *F. virguliforme*. Putative orthologous GH11 xylanases and GH28 PGs were determined at E value 10^-50^. MUSCLE in MEGA6 was used for protein sequence alignment [47]. SignalP 4.1 was used to detect secretory signal peptide [48]. SWISS-MODEL and QMEAN [49–52] were used to generate and evaluate a homology model for FvXyn11A (QMEAN6: 0.675) and FvXyn11B (QMEAN6: 0.708) based on *Chaetomium thermophilum* GH11 xylanase model 1h1a [53]. The protein-protein docking was performed by ZDOCK [54, 55]. The residue, E85 of *P. funiculosum* XYNC, E114 of FvXyn11A, and E98 of FvXyn11B, was set as indispensable interacting residuals with R179 of XIP-I and the modeling result was compare to interaction model 1te1 [5].

### Expression analysis of GH11 xylanases and GH28 PGs in vitro and *in planta*

In vitro RNA-Seq transcriptome was downloaded from DDBJ/EMBL/GenBank accession GBJV00000000 and analyzed as previously described [38]. HTSeq (version 0.6.1) were applied to quantify mapped reads for each transcript [56]. Transcripts with less than 60 reads across six libraries were filtered out in R (version 3.0.1) [57]. A false discovery rate of 0.05 was used as significant cutoff in edgeR analysis (version 3.6.4) [57–60]. Quantitative reverse-transcription polymerase chain reaction (qRT-PCR) was used to measure gene expression during root infection. Soybean seeds were germinated for 5 days at 25 °C. Each radicle was inoculated with 15 μl of 1× 10^6^ macroconidia per ml of *F. virguliforme*, and then incubated without light at 25 °C for 5 days before extracting total RNA by using TRIzol. Random primers were used to synthesize cDNA. Amplification efficiency of primers for qRT-PCR was determined based on four replicates and each replicate contained three concentration gradients (Table 3). Platinum® SYBR® Green qPCR SuperMix-UDG kit (Life Technologies) and Agilent Mx3005P qPCR System (Agilent Technologies) were used for qRT-PCR experiments. –ΔΔCt method was used to evaluate the expression of each gene [61] and gene expression was normalized to the translation elongation factor 1A of *F. virguliforme* (FvEF1A) [38, 62]. *In planta* gene expression analysis was repeated three times with three biological replicates for each. Statistics were conducted in R. Box-Cox power transformation was applied on raw data to fulfill the normal distribution of residuals. ANOVA and TukeyHSD were used to determine significance at *p* < 0.05.

## Abbreviations

AA, auxiliary activity; CAZy, carbohydrate-active enzymes; CBM, carbohydrate-binding module; CE, carbohydrate esterases; GH, glycoside hydrolases; GT, glycosyl-transferases; PCWDEs, plant cell wall degrading enzymes; PG, polygalacturonase; PGIP, polygalacturonase-inhibitor protein; PIPs, plant cell wall degrading enzyme inhibitor proteins; PL, polysaccharide lyases; SDS, sudden death syndrome; TAXI-III, *Triticum aestivum* xylanase inhibitor III; XIP-I, xylanase inhibitor protein I

## Additional file

Additional file 1: Table S1. CAZy of *Fusarium virguliforme*. (XLSX 480 kb)
