# Erratum to: *Genomic characterization of plant cell wall degrading enzymes and* in silico *analysis of xylanases and polygalacturonases of Fusarium virguliforme*

**DOI:** 10.1186/s12866-017-1020-8

**Published:** 2017-05-10

**Authors:** Hao-Xun Chang, Craig R. Yendrek, Gustavo Caetano-Anolles, Glen L. Hartman

**Affiliations:** 1 Department of Crop Sciences, University of Illinois, Urbana, IL 61801 USA; 2 Institute for Genomic Biology, Urbana, IL 61801 USA; 3 USDA-Agricultural Research Services, Urbana, IL 61801 USA; 4 National Soybean Research Center, University of Illinois, 1101 W. Peabody Dr., Urbana, IL 61801 USA

## Erratum

Upon publication of the original article [1], the title of this article read “*Genomic characterization of plant cell wall degrading enzymes and in silico analysis of xylanses and polygalacturonases of Fusarium virguliforme".* However, this was incorrect and the title of the manuscript should read “*Genomic characterization of plant cell wall degrading enzymes and in silico analysis of xylanases and polygalacturonases of Fusarium virguliforme*”. This has since been acknowledged and corrected in this erratum.
